# Sources of Fallacy in Regard to the Results of Treatment by Climate

**Published:** 1899-06-10

**Authors:** 


### SOURCES OF FALLACY IN REGARD TO THE RESULTS OF TREATMENT BY CLIMATE.

A consideration of the facts detailed by Sir Hermann Weber in his address to the Congress is sufficient to show how many fallacies may creep into any conclusions drawn from mere statistics in regard to the result of climatic or sanatorium treatment in such a malady as tuberculosis. It is admitted on all hands that certain groups of cases should not be sent to certain localities. Thus, in regard to high altitudes it is advised that cases with albuminuria, with advanced diabetes, with tuberculosis of the larynx, with extensive disease of one or both lungs—all of them serious conditions of bad prognostic augury—should not, as a rule, be sent into the mountains, while cases with limited disease and a strong constitution are looked upon as especially fitted for such places. The effect which such a selection of cases is likely to have upon statistics is obvious. Take, again, the different sanatoria. Some insist on a *régime* which practically drives away, or at any rate deters from entry, all except those who have a "strong constitution," much, no doubt, to the improvement of their statistics, while those which make themselves more attractive to patients of feeble build, although probably doing just as much, if not more good, are likely to suffer when judged by crude statistical methods. The establishment of sanatoria by industrial insurance associations muddles up the statistics in the same way. The whole aim of such institutions is to fit their patients for work, and only such cases as are likely to be fitted for work are taken in. The result is obvious enough to those who visit these institutions, for the patients are found to be well-looking men, whom no one would suppose to be suffering from pulmonary tuberculosis, very different from the patients we see in our hospitals for consumption. When we hear of statistical comparisons being drawn between different localities, different altitudes, and different sanatoria, we must not forget the very different cases which, as the result of a process of natural selection, are sent to them.

